# Personalized intrahepatic cholangiocarcinoma prognosis prediction using radiomics: Application and development trend

**DOI:** 10.3389/fonc.2023.1133867

**Published:** 2023-03-23

**Authors:** Pengyu Chen, Zhenwei Yang, Haofeng Zhang, Guan Huang, Qingshan Li, Peigang Ning, Haibo Yu

**Affiliations:** ^1^ Department of Hepatobiliary Surgery, Henan University People’s Hospital, Henan Provincial People’s Hospital, Zhengzhou, China; ^2^ Department of Hepatobiliary Surgery, People’s Hospital of Zhengzhou University, Zhengzhou, China; ^3^ Department of Hepatobiliary Surgery, Henan Provincial People’s Hospital, Zhengzhou, China; ^4^ Department of Radiology, People’s Hospital of Zhengzhou University, Zhengzhou, China

**Keywords:** radiomics, intrahepatic cholangiocarcinoma, deep learning, precision medicine, prognosis

## Abstract

Radiomics was proposed by Lambin et al. in 2012 and since then there has been an explosion of related research. There has been significant interest in developing high-throughput methods that can automatically extract a large number of quantitative image features from medical images for better diagnostic or predictive performance. There have also been numerous radiomics investigations on intrahepatic cholangiocarcinoma in recent years, but no pertinent review materials are readily available. This work discusses the modeling analysis of radiomics for the prediction of lymph node metastasis, microvascular invasion, and early recurrence of intrahepatic cholangiocarcinoma, as well as the use of deep learning. This paper briefly reviews the current status of radiomics research to provide a reference for future studies.

## 1 Introduction

After hepatocellular carcinoma, intrahepatic cholangiocarcinoma (ICC) is the second most frequent primary liver cancer (1). According to different classification methods, the least frequent type of bile duct cancer is intrahepatic cholangiocarcinoma, a malignant tumor that develops in the epithelial cells of the intrahepatic bile ducts, either in the small intrahepatic bile ducts or in the bile ducts close to the bifurcation of the hepatic ducts (2). According to epidemiological studies, ICC morbidity and mortality have recently increased globally (3, 4). Because there are no special clinical manifestations in the early stage of the disease, the majority of patients are found by chance during physical examination. Unfortunately, the prognosis of ICC patients with either surgical or non-surgical treatment is not satisfactory (5).

Currently, the most commonly used staging system for ICC is the AJCC TNM staging system, but this system was not specifically developed for postoperative prognostic prediction. Its accuracy may be compromised when used to predict the prognosis of patients undergoing a partial hepatectomy. In terms of clinical characteristics, imaging presentation, and therapeutic approach, ICC represents a distinct and habitual malignancy from hilar and distal biliary cholangiocarcinoma. A special model for prognostic prediction is necessary. Numerous researchers have created models that can objectively and accurately predict the prognosis of patients following ICC, and these established models have superior discriminative power and accuracy compared to the 8th version of the AJCC TNM staging system (6, 7). A useful predictive model can assist clinicians in selecting the most effective treatment strategy based on the individual prognosis of each patient. For clinicians, this is critical.

For preoperative tumor staging and resectability assessment of intrahepatic cholangiocarcinoma, cross-sectional computed tomography (CT) and magnetic resonance imaging (MRI) are the most commonly used imaging modalities (8). Physicians are able to use image information to refine treatment ideas. However, for patients with atypical imaging characteristics, physicians need to combine the advantages of multiple imaging techniques to obtain additional information from multimodal images. In fact, the full potential of medical imaging has failed to be realized in the clinical diagnosis and treatment process.

**Figure 1:**
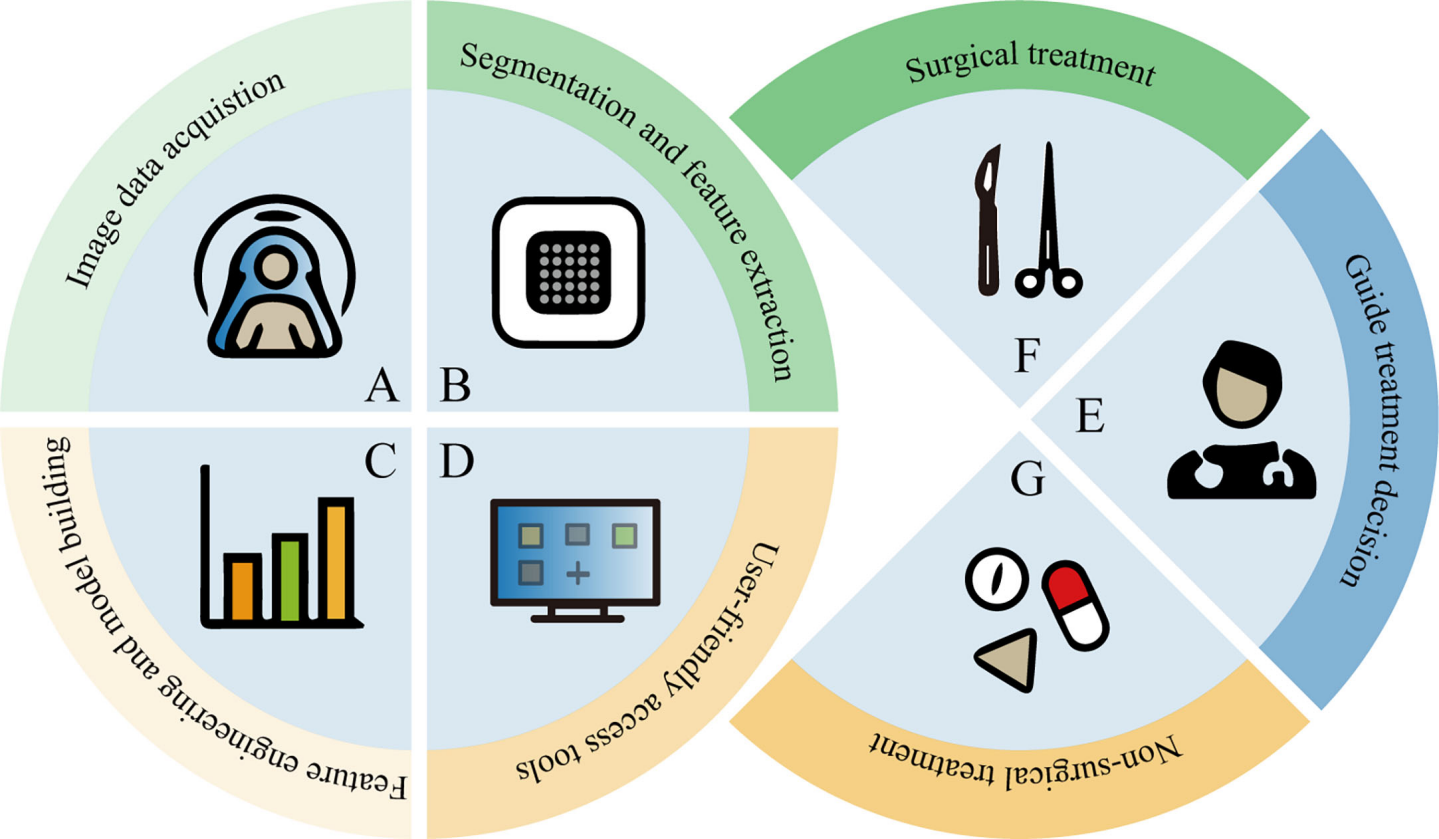
Radiomics model development and application process.

The development of high-throughput methods that can automatically extract a large number of quantitative imaging features from medical images for better predictive or diagnostic performance has been a subject of great interest (9). The application of radiomics and deep learning in this field refers to the feature extraction and quantitative analysis of image data from different modalities for the purpose of diagnosis and prediction. Radiomics begins with the acquisition of high-quality images, from which the radiologist then identifies, segments a region of interest (ROI) containing the entire tumor or tumor subregions, and ultimately presents it in two or three dimensions. Quantitative features (such as shape, grayscale, texture and wavelet features) are extracted from these ROIs, and a large number of features are filtered by feature engineering and placed in the database together with other data (such as clinical and genomic data). This data is then mined to develop predictive or prognostic models. These models are then represented by an interface-friendly tool that can be used by clinicians and guide clinical decisions (**Figure 1**). Deep learning features are learned automatically during the training process, which is distinct from conventional radiomics. And deep learning can be used for end-to-end modeling. Although these prediction models have not yet been applied in clinical practice, the advantages of this approach are to be affirmed.

Radiomics has received a lot of attention since it was proposed in 2012 (10). Traditional imaging can only capture simple semantic features, and the potential to reflect tumor heterogeneity is limited. However, radiomics approaches can provide a wealth of important complementary data which can help to uncover potential relationships between quantitative image features and heterogeneity. Today, the development of radiomics for tumor diagnosis, treatment decisions and prognosis prediction is encouraging, and numerous investigators are applying this approach to the field of intrahepatic cholangiocarcinoma.

## 2 Application of conventional radiomics in intrahepatic cholangiocarcinoma

### 2.1 Prediction of lymph node metastasis (LNM)

Lymph node metastasis is significantly associated with a poor prognosis in patients with intrahepatic cholangiocarcinoma (11). Currently, the prediction accuracy of lymph node status evaluation methods is usually unstable. Cross-sectional imaging alone can suggest the presence of lymph node metastases, but with limited identification (12, 13). Fine needle aspiration is invasive and has a limited role in detecting minor lymph node metastases (14). PET/CT is more sensitive than CT and MRI in detecting lymph node metastases, but its applicability seems fewer satisfactory for lymph nodes smaller than 1 cm (15). Histopathology is the gold standard for identifying the status of lymph nodes, but this strategy is only available in the postoperative period.

The development and validation of a radiomics model for preoperative prediction of lymph node metastasis in intrahepatic cholangiocarcinoma, based on the feasibility of the radiomics approach to evaluate lymph node status in ICC patients, can facilitate clinical decision-making and define the subgroup of patients who will benefit most from surgery. In one study (16), image features were extracted from arterial phase CT images to develop a radiomics model incorporating radiomics features and various risk factors for predicting lymph node metastasis. This model provides a powerful diagnostic tool for predicting lymph node metastasis and differentiates patients into high-risk and low-risk groups for lymph node metastasis likelihood, with significant differences in overall survival time and recurrence-free survival between the two groups. However, this study only extracted features from arterial phase CT images and the model was built on data from a single center and has not been validated in any external cohort. In another study (17), the fusion model of CT features was constructed by combining radiomics features of multiple sequences of CT images. In three cohorts (model training cohort, internal validation cohort, and external validation cohort), the area under the curve (AUC) values of the fusion model outperformed any radiomics model for monophasic CT images. The nomogram constructed in this study has demonstrated favorable differential and prognostic values through independent external validation.

This approach is not only feasible in CT, but also applicable in MRI. Xu et al (18). extracted image features from T1-weighted contrast-enhanced MRI and used a support vector machine (SVM) classifier to construct a prediction model after filtering radiomics features. Combining SVM scores and two clinical features (CA19-9 levels and MR-reported LNM factors) to construct a combined nomogram, both the ROC curve and the decision curve suggested that the constructed model had decent performance. Numerous studies have reported a strong correlation between LNM and ICC prognosis, making this non-invasive method a potential preoperative assessment and prognostic assessment tool for ICC due to its great predictive ability of preoperative LN status.

### 2.2 Prediction of microvascular invasion (MVI)

Microvascular invasion is an independent risk factor for prognosis in patients undergoing radical ICC resection (19). With the increasing understanding of MVI and its prognostic value, the prediction of MVI has become a focus of research in recent years, both in hepatocellular carcinoma and intrahepatic cholangiocarcinoma (20–22). Moreover, some qualitative or quantitative images were found to be correlated with MVI (23). Clinical microvascular invasion is primarily detected by histopathology under a microscope. However, due to the heterogeneity of the tumor and the sampling error, the preoperative biopsy results may be unreliable. The radiomics approach addresses this concern by allowing features to be extracted throughout and around the tumor.

Peng et al (24). study to develop a radiomics model based on ultrasound (US) medical images and the first study to use an ultrasound radiomics approach to non-invasively assess MVI. This study showed the feasibility of using radiomics methods to predict microvascular invasion of ICC, but the performance of the model seems to be less satisfactory. Since then, the vast majority of research has been devoted to the improvement of model performance. Zhou et al (25). developed a radiomics model based on dynamically enhanced MRI images with AUC of 0.873 and 0.850 in training and validation cohorts, respectively. This model can be useful for pre-operative prediction of microvascular invasion in patients with mass-like intrahepatic cholangiocarcinoma and is instructive for individualized treatment of ICC patients. Another study (26) used recursive feature elimination support vector machine to construct the radiomics model, combining radiological factors and radiomics features to construct nomogram with an AUC of 0.886 for the training dataset and 0.80 for the test dataset, achieving satisfactory performance. Qian et al (27). extracted radiomics features from six sequences (DWI, T2WI-FS, 3DVIBE T1WI, AP, PVP, and DP) of images that could help predict the MVI status of ICC patients. The construction of nomogram for predicting MVI (containing: combined radiomics features, clinical features and imaging features) had an AUC of 0.953 for the training cohort and 0.861 for the validation cohort, further improving the predictive performance of the MVI diagnosis. In addition, the study also compared logistic regression (LR), random forest (RF) and SVM classifier modelling effects, respectively. The aim of the study by Jiang et al (28). was to investigate the contribution of 18f -fluorodeoxyglucose positron emission tomography/computed tomography (18F-FDG PET/CT) radiological features to MVI. The results suggest that the PET model has better performance than the CT model or PET combined CT model in ICC MVI prediction. Fiz et al (29). elucidated that radiomics features of the tumor and peritumor region extracted from preoperative PET/CT could provide noninvasive biological characterization of ICC. For MVI prediction, the combined clinical-radiomics model was superior to the preoperative clinical model alone and achieved a performance no less than that of the postoperative pathological model. It can be seen that it is valuable to predict MVI on the basis of preoperative images, which can help doctors to make clinical decisions related to treatment plans and influence the choice of individualized treatment plans.

### 2.3 Prediction of early recurrence (ER) of postoperative patients

Partial hepatectomy is the preferred option for radical treatment of ICC, yet postoperative survival remains unsatisfactory and the main reason for the poor prognosis is the high recurrence rate (30). Understanding the timing associated with recurrence is expected to inform discussions around adjuvant therapy, monitoring and treatment of recurrent disease (31). The qualitative and quantitative features of the images have predictive value for postoperative recurrence time of intrahepatic cholangiocarcinoma (32). But reliably identifying patients at high risk for ER remains a challenge, hindering the decision-making process for personalized treatment of ICC patients. Although some predictive models constructed based on clinical characteristics exist, they are limited by poor accuracy and cannot be widely used. This has led clinicians to seek safe, effective and novel methods to identify high-risk patients for ER. Medical imaging plays an important role in the preoperative evaluation of ICC, so it is necessary to find an image-based method to predict the early recurrence of ICC. Many studies have done the same, of course.

The optimal threshold for distinguishing between early and late recurrence after surgery for intrahepatic cholangiocarcinoma is 24 months (33). Liang et al (34). constructed a model that combines radiomics features and clinical staging to predict early recurrence in ICC patients undergoing a partial hepatectomy. This is the first study to use MRI features to predict ER in intrahepatic cholangiocarcinoma, and the AUCs of the training and validation team nomograms were 0.90 and 0.86, respectively, and the decision curve analysis confirmed the value of this model for clinical application. Zhao et al (35). developed MRI radiomics models (AP, PVP, DP), clinicopathology-imaging models (CPR), and combined models. The performance of the three models for detecting early recurrence in patients after ICC was compared, and the results obtained were that the combined model had a stronger predictive performance (AUC=0.949). Moreover, this study points out the most important feature parameters for predicting ER, mainly the texture features from MRI AP sequences. To investigate the value of peritumoral area in predicting early recurrence of ICC. Xu et al (36). combined quantitative magnetic resonance imaging features based on intratumoral and peritumoral (3 or 5 mm) regions. Among all early recurrence prediction models, the combined radiomics-clinical model with intratumoral and 5-mm peritumoral regions performed the best.

However, we also found that the time of early recurrence was different according to the definition of different investigators, and further study is needed to determine the optimal cut-off value of early and late recurrence. Yang et al (37). developed a prediction model based on diffusion-weighted imaging based on recurrence within 1 year after surgery as early recurrence. A multivariate logistic regression analysis was used to construct a comprehensive nomogram and a CPR model was developed as a reference to demonstrate the incremental value of radiomics features in predicting ER. Low- and high-risk groups defined according to this model helped to identify patients who might benefit from postoperative adjuvant chemotherapy for ICC, improving prognostic prediction. Zhu et al (38). regarded relapse 6 months after surgery as early recurrence. The model was established according to the CT image features, radiomics features, clinical indicators and pathological features of mass-forming ICC. As in the previous study, the combined model performed better. To explore the impact of classifier selection on prediction model performance. Hao et al (39). performed a CT-based radiomics analysis in a three-center cohort, using multiple feature selection algorithms and classifiers for modeling. A more generalized model was selected to predict ER in ICC patients. Observation of the above studies for predicting ER of intrahepatic cholangiocarcinoma found that multicenter retrospective and prospective verification should be carried out in follow-up studies to obtain a higher level of evidence.

Application of radiomics in predicting lymph node metastasis, microvascular invasion, and early recurrence (**Table 1**).

**Table 1:** Application of radiomics in predicting lymph node metastasis, microvascular invasion, and early recurrence.

Ref	Year	Type*	Image	Population	Number of cases	Segmentation (ROI)	Feature extraction	Feature selection + Classifier **	Performance			
									Accuracy	Sensitivity	Specificity	AUC
Liang et al	2018	C	MRI	Single center	209	Manually	467	LASSO; LR		0.74	0.89	0.90 (0.83–0.94)
Ji et al	2019	A	CT	Single center	155	Manually	105	LASSO; LR		0.868	0.736	0.846 (0.768-0.925)
Xu et al	2019	A	MRI	Single center	148	Manually	491	mRMR; SVM	0.7264	0.8936	0.5763	0.842 (0.758-0.906)
Zhao et al	2019	C	MRI	Single center	47	Manually	396/phase	Hypothetical test; LR	0.87	0.94	0.84	0.95 (0.90-1.00)
Peng et al	2019	B	US	Single center	128	Manually	1076	Hypothetical test; SVM	0.848	0.550	0.933	0.699
Zhou et al	2021	B	MRI	Single center	126	Manually	788/phase	LASSO; LR	0.863	0.750	0.906	0.873 (0.796-0.950)
Xu et al	2021	C	MRI	Single center	209	Manually	2268	mRMR; bootstrap		0.74	0.78	0.825 (0.756−0.880)
Zhu et al	2021	C	CT	Single center	125	Manually	87/phase	LASSO; LR	0.877	0.833	0.892	0.917 (0.840-0.965)
Hao et al	2021	C	CT	Three centers	177	Manually	473	mRMR; GBM	0.750	0.879	0.603	0.802 (0.727-0.876)
Xiang et al	2021	B	CT	Single center	157	Manually	1130/phase	Hypothetical test; SVM	0.845	0.771	0.903	0.886 (0.823–0.949)
Qian et al	2022	B	MRI	Two centers	187	Manually	2600/phase	LASSO; LR/RF/SVM	0.892/0.946/0.869	0.974/0.895/0.763	0.859/0.967/0.913	0.953/0.988/0.898
Fiz et al	2022	B	PET/CT	Single center	74	Manually		Backward stepwise/PCA	0.819	0.853	0.789	0.881
Yang et al	2022	C	MRI	Single center	124	Manually	110	mRMR; RF; LR	0.76	0.77	0.73	0.82 (0.68–0.96)
Zhang et al	2022	A	CT	Two centers	296	Manually	832	mRMR; DT; LR	0.94	0.87	0.97	0.98 (0.96–0.99)
Jiang et al	2022	B	PET/CT	Single center	127	Semi-automatically	1815	Hypothetical test; RF	0.77	0.75	0.80	0.90
Gao et al	2022	B	MRI	Three centers	519	CNN			0.92	0.94	0.91	0.98 (0.96–0.99)
Wakiya et al	2022	C	CT	Three centers	41	CNN			0.98	0.99	0.97	0.998

ROI, regions of interest; AUC, area under curve; LASSO, least absolute shrinkage and selection operator; LR, logistic regression/linear regression; mRMR, maximal relevance and minimal redundancy; SVM, support vector machine; GBM, Gradient Boosting Machine; RF, random forest; PCA, principal components analysis; DT, decision tree; CNN, convolutional neural network; *, A = Prediction of lymph node metastasis; B = Prediction of microvascular invasion; C = Prediction of early postoperative recurrence in patients. **, These studies used a variety of feature selection methods and classifiers. This column shows only the main methods described in the paper.

### 2.4 Prediction of overall survival time (OS)/progression-free survival (PFS) before operation

Currently, the most commonly used staging system for ICC is the tumor, lymph node and metastasis based TNM staging, which is based on only a limited number of tumor features. Due to tumor heterogeneity, the prognosis of ICC patients varies from individual to individual and its predictive accuracy is somewhat limited (40, 41). In addition, many prognostic models for intrahepatic cholangiocarcinoma rely on surgical pathology data and are not applicable in a preoperative setting. This shows the need for enhanced risk stratification to better predict clinical outcomes and optimize perioperative management. In clinical practice or in future clinical trials, good adjunctive prognostic prediction tools such as radiomics-based prediction models and nomograms have been proposed. This is urgently needed to develop treatment strategies.

For the above reasons, it was also to verify the value of radiomics in preoperative prediction of the prognosis of ICC. Silva et al (42). developed a prognostic model that combines clinical parameters and radiomics features to test the predictive power of radiomics through a unique scale for survival stratification. Another study (43) investigated the predictive value of an enhanced CT-based radiomics model for the prognosis of intrahepatic cholangiocarcinoma. The covariates in the nomogram include radiomics scores and some clinical indicators. Park et al (44). aimed to develop and validate a preoperative model capable of predicting postoperative outcomes. Three different models are constructed using clinical, radiological, and radiomics features, respectively. The study focused on predicting recurrence-free survival and the clinical-radiological-radiomics model performed optimally with a training group c-index of 0.75 (0.72-0.79). This facilitates the preoperative assessment of postoperative outcomes in patients with mass-forming ICC in order to select the best treatment option at the time of the initial treatment decision. Deng et al (45). developed a nomogram to assess the prognosis of radical resection in ICC patients. It is a clinical-radiomics model that combines sarcopenia, additional clinical features and radiomics scores. Similar to previous studies, the c-index of the clinical-radiomics model was 0.766, which was significantly higher than the c-index of the different models: 0.667 (radiomics model), 0.598 (tumor differentiation system) and 0.563 (AJCC 8th edition), respectively. Furthermore, the clinical-radiomics model predicted 1 and 3-year OS with an AUC of 0.809 and 0.886, respectively, which was the most accurate prognostic prediction for patients with ICC with mass formation.

Due to the lack of ultrasound and PET radiomics studies related to ICC prognosis. Li et al (46). were the first to extract radiomics features from baseline US and CEUS images (four sequences) to construct a preoperative model for predicting OS in patients with intrahepatic cholangiocarcinoma. The C-index of nomogram containing CA 19-9, gender, ascites, radiomics features and radiological features was higher than the 8th TNM staging system. It was also found that the predictive efficacy of radiomics alone was not statistically superior to TNM staging. Fiz et al (29). compared a preoperative radiomics model for PET/CT imaging with a model for postoperative pathological data in order to assess the prognosis of patients with ICC with masses who underwent preoperative hepatectomy. Both models have similar performance in predicting patient OS/PFS, which further demonstrates the predictive power of radiomics for prognostic assessment. Another study (47) used MRI-based radiomics models to predict pre-operative survival outcomes in patients. The integration of radiomics features into the TNM staging system significantly improves the accuracy of prognosis prediction compared to TNM staging alone.

Application of radiomics in predicting OS/PFS (**Table 2**).

**Table 2:** Prediction of overall survival time/progression-free survival before operation.

Ref	Year	Type	Population	Number of cases	Segmentation	Feature extraction	Feature selection	C-index
Silva et al	2021	CT (Portal venous phase)	Single center	78	Manually		PCA	0.81
Tang et al	2021	CT (Portal venous phase)	Single center	101	Manually	42	LASSO	0.783/0.751
Park et al	2021	CT (Two sequences)	Six centers	345	Manually	661	LASSO	0.75 (0.72-0.79)
Deng et al	2021	CT (Two sequences)	Single center	82	Manually	214	Cox + AIC	0.768 (0.765-0.770)
Li et al	2022	US (Four sequences)	Single center	170	Manually	1044/phase	LASSO	0.72/0.75
Fiz et al	2022	PET/CT	Single center	74	Manually		correlation/PCA	0.80/086
Yang et al	2022	MRI (Six sequences)	Single center	163	Manually	4998	LASSO	0.750 (0.680-0.819)

PCA, principal components analysis; LASSO, least absolute shrinkage and selection operator; AIC, Akaike information criterion.

### 2.5 Other applications

Identifying tumors can guide treatment and make more appropriate choices. It is sometimes difficult to identify intrahepatic cholangiocarcinoma in a non-invasive manner or to distinguish it from other tumors. Most ICC and hepatocellular carcinoma (HCC) lesions have a similar imaging presentation. Qualitative image analysis is not sufficient to differentiate between HCC or ICC. Xu et al (48). developed a diagnostic model based on CT images to provide a reference for future differential diagnosis of HCC and ICC. Its diagnostic power is superior to that of experienced radiologists. Ren et al (49). aimed to explore machine learning-based ultrasomics in the preoperative noninvasive identification of hepatocellular carcinoma and intrahepatic cholangiocarcinoma. The combined model has a strong performance, yet the performance of the ultrasomics model alone is poorer than the clinical model. Another study on MRI-based radiomics nomogram models also showed excellent discriminatory performance (50). This approach is not limited to the differentiation of ICC from HCC. Some studies have also been able to distinguish intrahepatic cholangiocarcinoma well from combined hepatocellular and cholangiocarcinoma (51, 52), hepatic lymphoma (53), and inflammatory mass with hepatolithiasis (54, 55). In addition, there may be differences between preoperative assessment and intraoperative findings in some patients. Chu et al (56). developed a model that accurately predicted preoperative ineffective excision to guide clinical work.

At present, the evaluation of tumor biological characteristics is mainly based on immunohistochemistry. In cancer patients, however, different parts of the tumor have different molecular signatures, and this variation can also change over time. Since it is not possible to biopsy every part of every tumor at multiple points in time, the assessment of the biology of a biopsy sample may not be representative of the whole tumor (57). Yet medical imaging contains a wealth of information about the tumor microenvironment and potential treatment outcomes. Peng et al (24). attempted radiomics approach to evaluate some immunohistochemical features: Ki-67, VEGF and CK7. Various data dimensionality reduction methods and machine learning algorithms are used to select the optimal model, and the model predictions achieve great results. It can be seen that radiomics can use the extracted feature information to characterize intra-tumor heterogeneity. Zhang et al (58). used preoperative magnetic resonance imaging texture analysis to predict the immunophenotype of patients with intrahepatic cholangiocarcinoma. Patient samples were classified into inflammatory or non-inflammatory immunophenotypes based on the density of CD8+ T cells, and logistic regression analysis was applied to select the significant features associated with immunophenotype. From the constructed immunophenotype prediction model, it was known that the inflammatory immunophenotype had a better prognosis than the non-inflammatory immunophenotype. It can be seen that MRI texture features can be used as biomarkers to predict immune phenotypes and clinical outcomes in ICC patients.

**Figure 2:**
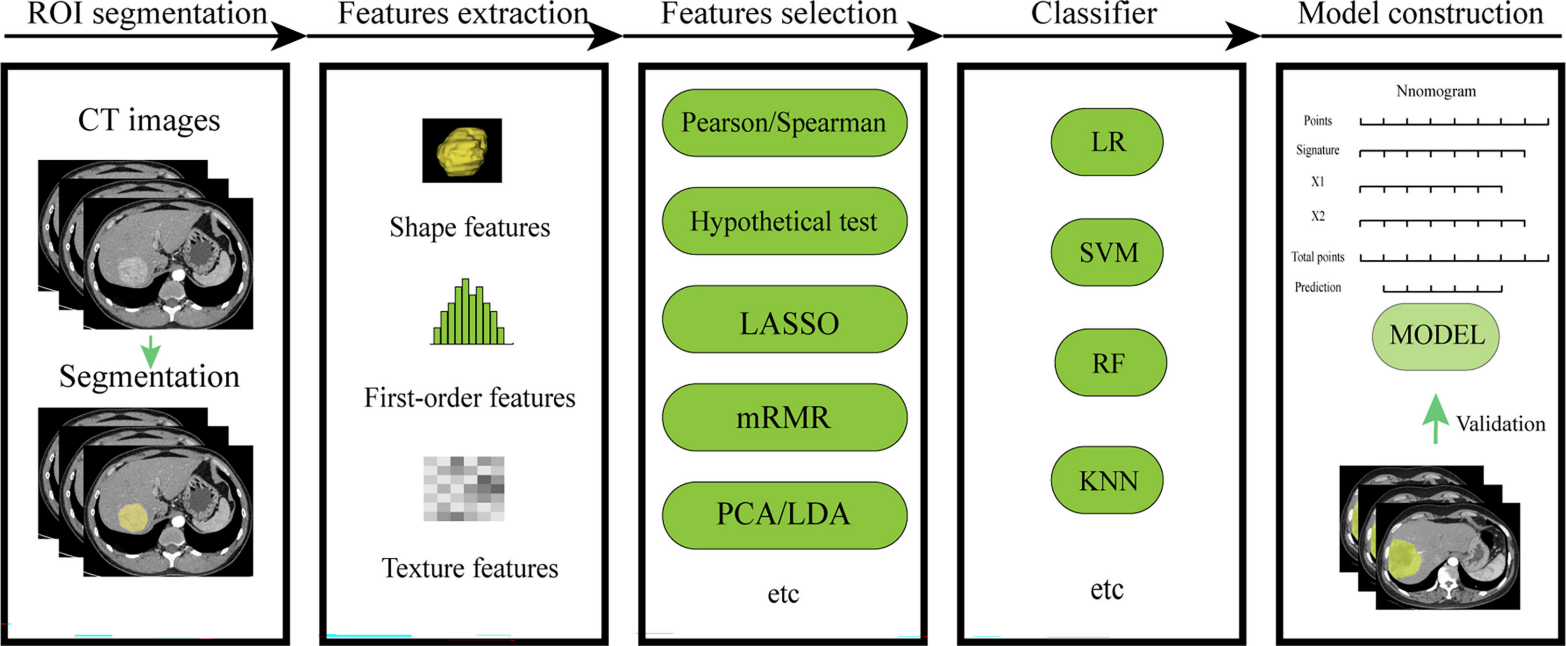
Workflow of conventional radiomics. LASSO, least absolute shrinkage and selection operator; mRMR, maximal relevance and minimal redundancy; PCA, principal components analysis;LDA, linear discriminant analysis; LR, logistic regression; SVM, support vector machine; RF, random forest; KNN, k-nearest neighbor.

Workflow of conventional radiomics (**Figure 2**).

## 3 Application of deep learning in intrahepatic cholangiocarcinoma

### 3.1 Deep learning for model construction

**Figure 3:**
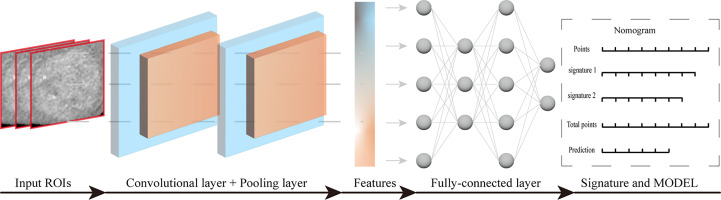
Workflow of deep learning for model construction.

In recent years, artificial intelligence algorithms, particularly deep learning (DL), have made significant advances in the field of medical image analysis, driving the field forward at a rapid pace (59, 60). Among them, convolutional neural network (CNN) is a commonly used deep learning method that has great potential in various clinical tasks, such as disease diagnosis and classification. It can analyze not only pathological images but also medical images. Deep learning-radiomics is a newly developed method that can extract a large number of valuable quantitative features from medical images. Moreover, the continued development of convolutional network architectures has helped to build more compact and accurate models (61). The introduction of deep learning allows radiomics to model medical data end-to-end and to perform multi-task learning for multiple clinical tasks. Good results have been achieved in studies of nasopharyngeal carcinoma (62), non-small cell lung cancer (63), and breast cancer (64). The basic structure of convolutional neural network includes: convolutional layer, pooling layer, fully connected layer and output layer. Convolutional layer and pooling layer were used for feature extraction and feature dimension reduction, respectively. The fully connected layer and the output layer can integrate the features and finally complete the classification. Workflow of deep learning for model construction (**Figure 3**).

Current studies also exist on deep learning models constructed for predicting early ICC recurrence, microvascular infiltration. Gao et al (65). developed a DL model based on DCE-MRI multiparametric fusion for preoperative assessment of ICC MVI. The DL model allows for complementary feature extraction and cross-scale fusion of multiple MRI sequences, showing better performance than traditional radiomics models for MVI status in ICC patients. In the Wakiya et al (66). study, early recurrence was defined as recurrence within 1 year after liver surgery. No previous application of DL to ICC post-resection recurrence prediction has been reported. This study developed a prediction model using CNN construction that successfully demonstrated strong performance in predicting early postoperative recurrence using preoperative CT images. The model can directly predict early recurrence and predict who should receive adjuvant chemotherapy based on the risk of recurrence, presenting a novel perspective on ICC management. Of course, there are also some tumor identification studies, which show the feasibility of deep learning system to identify ICC, and the simultaneous identification of more types of lesions is becoming a trend (67–69). Its discrimination ability is superior to that of a radiologist, and it can even combine the experience and intuition of a radiologist with the computational power of a DL decision support tool to optimize workflow and obtain higher quality diagnostic results (70).

### 3.2 Deep learning for feature extraction

**Figure 4:**
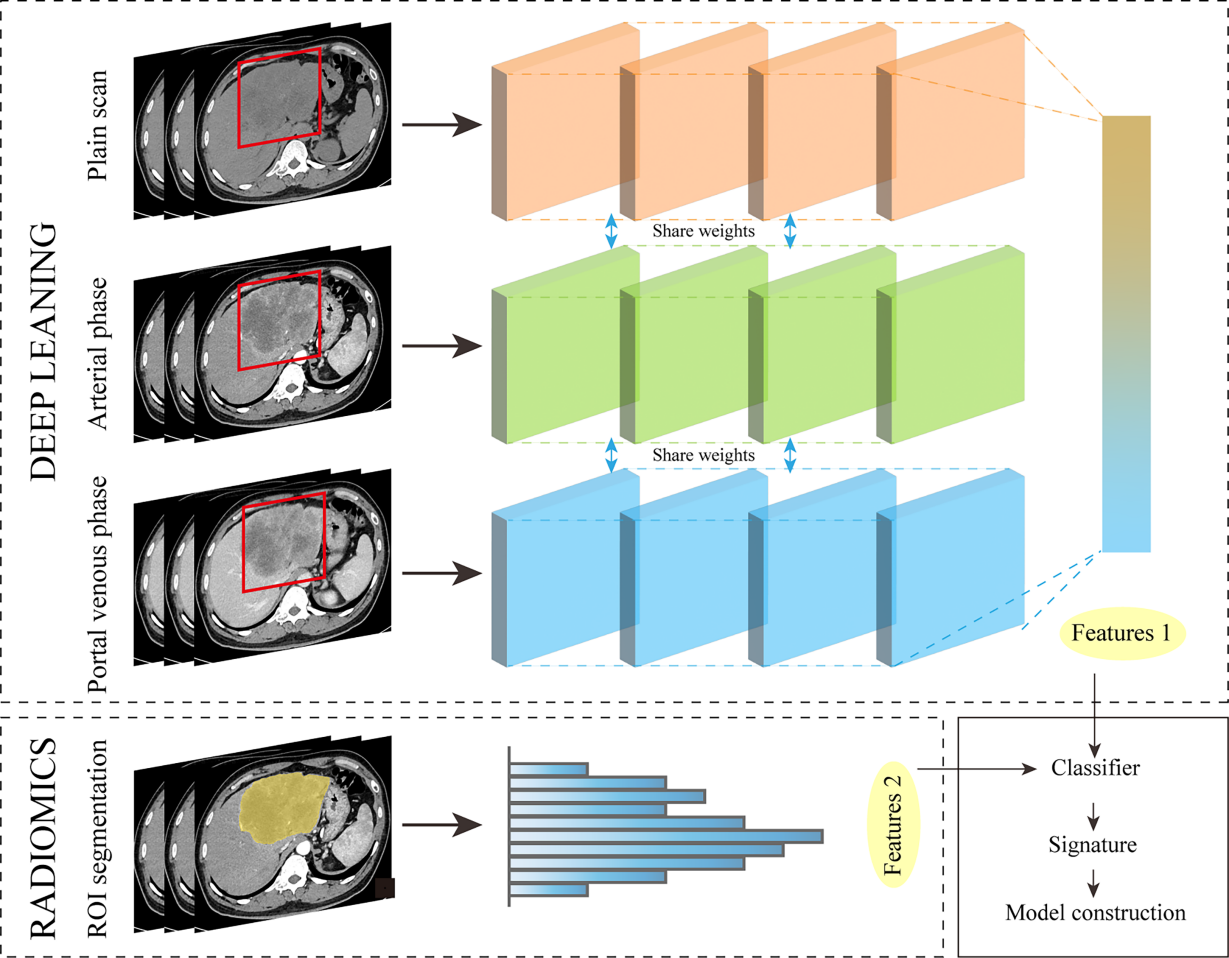
Workflow of deep learning for feature extraction.

In some studies on gastric cancer (71), COVID-19 pneumonia (72), and hilar cholangiocarcinoma (73), models constructed from a combination of features extracted from deep learning methods and radiomics features have shown strong performance. It can be seen that applying this method to the study of ICC will also achieve good results. Preoperative models based on AI and human collaboration in imaging can provide substantial benefits for treatment decisions and can be easily and non-invasively applied prior to surgery. Workflow of deep learning for feature extraction (**Figure 4**).

However, these deep learning features are not specifically defined, and future work should explore the image encoding process used to generate each deep learning feature to further enhance the interoperability of these features. Wang et al (74). enabled radiologists to interpret the decision elements behind classification decisions through automatic recognition, mapping, and scoring of radiological features by a DL system. Despite the lack of validation criteria for feature maps and correlation scores, this fuels the integration of deep learning into clinical practice. ()

## 4 Advantages and disadvantages of conventional radiomics and deep learning

(1) In conventional radiomics, machine learning approaches are fast to run, good for small-scale data, and easy to understand and implement. However, overfitting of the model can occur.

(2) For deep learning, data enhancement can be achieved by transforming medical images (e.g., rotating angles, moving, flipping, and scaling), and the neural network architecture can be flexibly tuned. The overfitting phenomenon can be avoided while improving the model performance. However, this approach often sacrifices the interpretability of the model. In clinical work, clinicians are often not happy to use tools that are difficult to understand.

(3) In conventional radiomics, the process of outlining ROIs is often time-consuming, and the accuracy of manual outlining has been questioned. But in the end-to-end CNN architecture, there is no need to segment the lesions precisely.

## 5 Trends in radiomics

### 5.1 Radiogenomics

Although radiomics has been used in oncology for more than a decade, its potential has not been fully tapped. Yu et al (75). explored the relationship between radiomics features and the tumor microenvironment in breast tumors, and key features were significantly and linearly correlated with immune cells, long noncoding RNA and methylation sites. Changes in gene expression are strongly associated with some imaging traits, and this relationship is not accidental (76). The systematic link between imaging features and gene expression allows for useful inferences from both directions, using visual images to translate gene expression. This approach represents an emerging field called radiogenomics. The diversity of today’s imaging modalities (CT, MRI, US and PET) and the easier access to high-resolution images provide favorable conditions for research in this direction. In a multicenter study (77), radiogenomics was used to predict MVI in liver cancer. Although this study was based on qualitative imaging features only, good results were achieved. Diagnostic performance did not differ significantly between institutions or between tumor sizes. In a study of colorectal cancer, Yang et al (78). found that CT-based radiomics features were significantly associated with KRAS/NRAS/BRAF mutations. Hoivik et al (79). used a radiogenomic approach to integrate preoperative MRI with histology, transcriptomics, and molecular biomarkers to identify aggressive tumor features. A correlation between quantitative features of the tumor and gene expression was also shown in a study of intrahepatic cholangiocarcinoma (80). In addition, the use of clinically available images has the advantage of being inexpensive and easy to implement compared to gene sequencing. It is thus seen that this will be a direction of development for radiomics in predicting personalized prognosis of intrahepatic cholangiocarcinoma.

### 5.2 Guiding immunotherapy

In recent years, new insights into immunotherapy for oncology patients have emerged, and the reliable identification of ICC immunophenotypes is clinically important for predicting response to immune checkpoint blockade. For example, as blockade of the PD-1/PD-L1 pathway becomes more widespread in cancer therapy, understanding their expression status in ICC patients can help determine which patients are most likely to benefit and enable personalized treatment. Zhang et al (81). used radiomics features from MRI as a non-invasive biomarker to predict PD-1/PD-L1 expression in ICC patients. This predictive model can help clinicians assess the value of immunotherapy and make appropriate clinical decisions. It can also avoid unnecessary surgery for some ICC patients and avoid their harm and financial loss. Radiomic signatures can help to identify tumor immunotherapy phenomena, which will provide new theoretical and experimental foundations for precision medicine.

### 5.3 Virtual biopsy

The development goal of radiomics is “virtual biopsy”. The use of machine learning methods gradually failed to meet the demands of researchers. Currently, more and more studies use deep learning methods to build more accurate models. Radiomics has the potential to be used as a quantitative, non-invasive prognostic biomarker for clinical practice.

## 6 Discussion

From the observations of previous studies, we find some patterns and problems. (1) The choice of classifier is one of the influencing factors of model effectiveness. Some studies compare different classification methods to obtain the method with the best results in terms of certain predictions (39, 53). (2) Some studies suggest that the extracted radiomics features in arterial phase images have a better predictive performance. The reason for slightly better AP imaging than PVP may be related to the different arterial blood flow (51). (3) Models constructed with 3D image features have an advantage over 2D image feature models. This rule has at least been shown in studies of intrahepatic cholangiocarcinoma. This could also be related to the type of imaging data, the endpoint events of the predictive model, or the volume size of the tumor (82). (4) Standardization of image scans and protocols remains a challenge in the study of multiple sequences of images from different centers or different instruments. Without a uniform standard for image formats, the accuracy of modeling analysis will be compromised. Perrin et al (83). found that contrast agent injection rate and pixel resolution affect feature repeatability. (5) Many current studies are based on a single center and have limited sample size and lack prospective studies. Studies where a large number of radiomics features are extracted can lead to model overfitting and poor validation sets when the sample size is too small. (6) Radiomics models alone may not necessarily have better efficacy than clinical models, but considering their objectivity, constructing models in combination with clinical indicators will achieve reliable results. Observations of related studies in recent years have revealed that this is also the case (23). (7) Previous studies have found that most of the radiomics features extracted in ROI, after feature engineering screening, are texture features or wavelet texture features (24, 58). This pattern has been found not only in studies of ICC, but also in studies of other tumors. For example, in breast cancer studies (75), significant differences were found in key radiomics features of patients before and after neoadjuvant chemotherapy, and two wavelet features were significantly associated with chemotherapy response (wavelet-LLL-GLCM, wavelet-HHH-GLSZM). Wavelet features may have potential associations with pathophysiology, proteomics and tumor morphology that are not captured by clinicians through low-level radiomics features or visual inspection (34). (8) The segmentation methods of tumor images can be divided into three categories: manual segmentation, semi-automatic segmentation, and automatic segmentation. However, current research is still dominated by manual segmentation. There is no uniform conclusion as to which segmentation method is more standard. Manual and semi-automatic segmentation techniques are time-consuming, and the ROI outlined in differs between readers due to differences in shape, size, and boundaries. (9) The outline of the region of interest is no longer limited to the tumor itself, but can also include sub-regions of the tumor, for example, the peritumoral part also contains some valuable information. (10) At present, most studies are focused on improving the performance of the model, but relatively few studies have focused on the interpretability, which may be a direction for future research. (11) Studies are now not limited to CT and MRI images, but there are also some studies that have explored PET and US images. Radiomics-related research is moving toward plurality. However, non-redundancy and reproducibility are issues that need to be addressed in order to apply some of the research results to the clinic in the form of biomarkers.

## 7 Conclusion

Radiomics can extract features from multiple time sequences and images containing the entire tumor, which is of great significance to the research of tumor related fields. The modeling analysis using radiomics has achieved satisfactory results in predicting lymph node metastasis, microvascular invasion and early recurrence of intrahepatic cholangiocarcinoma. In addition, deep learning and radiogenomics will be the trends in research methods and research directions, respectively. With the advent of multi-omics and multi-modal research, applying this approach to the study of intrahepatic cholangiocarcinoma is not only a challenge, but also an opportunity.

## Author contributions

Conception of study: PC, ZY, and HY. Collecting literature: PC and ZY. Figures and tables: PC, ZY, and QL. All authors contributed to writing of the manuscript and approved the final manuscript. All authors contributed to the article and approved the submitted version.
